# Digital reconstruction of the Ceprano calvarium (Italy), and implications for its interpretation

**DOI:** 10.1038/s41598-017-14437-2

**Published:** 2017-10-25

**Authors:** Fabio Di Vincenzo, Antonio Profico, Federico Bernardini, Vittorio Cerroni, Diego Dreossi, Stefan Schlager, Paola Zaio, Stefano Benazzi, Italo Biddittu, Mauro Rubini, Claudio Tuniz, Giorgio Manzi

**Affiliations:** 1 Dipartimento di Biologia Ambientale, Sapienza Università di Roma, Roma, Italy; 2 Istituto Italiano di Paleontologia Umana, Roma, Italy; 3 Centro Fermi - Museo Storico della Fisica e Centro di Studi e Ricerche ‘Enrico Fermi’, Roma, Italy; 4 The ‘Abdus Salam’ International Centre for Theoretical Physics, Trieste, Italy; 5 Italian Ministry of Culture, Anthropological Service, Roma, Italy; 6 Electra - Sincrotrone Trieste, Trieste, Italy; 7 Department Biological Anthropology, University Medical Center, Freiburg, Germany; 8 Department of Cultural Heritage, University of Bologna, Bologna, Italy; 9 Department of Human Evolution, Max Planck Institute for Evolutionary Anthropology, Leipzig, Germany; 10 Dipartimento di Archeologia, Università di Foggia, Foggia, Italy; 11 Centre for Archaeological Science, University of Wollongong, Wollongong, Australia

## Abstract

The Ceprano calvarium was discovered in fragments on March 1994 near the town of Ceprano in southern Latium (Italy), embedded in Middle Pleistocene layers. After reconstruction, its morphological features suggests that the specimen belongs to an archaic variant of *H. heidelbergensis*, representing a proxy for the last common ancestor of the diverging clades that respectively led to *H. neanderthalensis* and *H. sapiens*. Unfortunately, the calvarium was taphonomically damaged. The postero-lateral vault, in particular, appears deformed and this postmortem damage may have influenced previous interpretations. Specifically, there is a depression on the fragmented left parietal, while the right cranial wall is warped and angulated. This deformation affected the shape of the occipital squama, producing an inclination of the transverse occipital torus. In this paper, after X-ray microtomography (μCT) of both the calvarium and several additional fragments, we analyze consistency and pattern of the taphonomic deformation that affected the specimen, before the computer-assisted retrodeformation has been performed; this has also provided the opportunity to reappraise early attempts at restoration. As a result, we offer a revised interpretation for the Ceprano calvarium’s original shape, now free from the previous uncertainties, along with insight for its complex depositional and taphonomic history.

## Introduction

The hominid fossil calvarium discovered near Ceprano (Frosinone, Latium, Italy) represents a rather puzzling specimen in relation to other European hominins of the Middle Pleistocene^1^. For a decade after the discovery (March 13th, 1994), its chronology was considered to be around 800–900 ka^2–4^, whereas more recent data points to the beginning of MIS 11, providing a date bracketed between 430 and 385 ka^5^. This revised chronology corresponds to a time span when European cranial and postcranial samples already exhibited derived phenotypes shared with *Homo neanderthalensis* ^6–10^. The absence of clear Neanderthal-like traits in the Ceprano calvarium^2–4^, combined with its unique combination of architectural and discrete features^4,11–13^, distinguishes the Italian specimen from great part of the penecontemporaneous fossil record in Europe, suggesting that its morphology probably persisted in an eco-geographic isolated *refugium*, while more derived morphs were spreading across the continent^1,14^.

From a taxonomic point of view, since its discovery the Ceprano calvarium has been alternatively viewed as a “late” *H. erectus* ^2^, a possible adult representative of *H. antecessor* ^4^ and even the holotype of a new species named *H. cepranensis* ^15^. By contrast, Ceprano is more probably representative of an archaic variant of *H. heidelbergensis* ^12^, furnishing evidence for the cranial morphology of the still poorly known stem subspecies (or paleodeme) of this taxon^1,16^.

## Background

The Ceprano calvarium was discovered by one of us (I.B.) in an area known as Campogrande, encompassed between the small towns of Ceprano, Pofi and Castro dei Volsci in southern Latium. The discovery occurred after a bulldozer sectioned (approximately from west to east) the clay containing the cranium and the other overlying sediments. Thus, the fragments of the calvarium were found within a stratified series of fluvio-lacustrine deposits^2–5^ and some of them were still *in situ*. Specifically, great part of the frontal bone was found with the brow ridges inclined downwards and inserted in the clay, showing the coronal profile (parallel to the east-west section of the deposit) and the endocranial surface to the observer, suggesting that the cranium was facing toward south-south-west before it was disturbed, with the rear portion of the vault rather upwards (I.B., personal observation). Approximately 50 large fragments were unearthed in a small area near the original find and more than 200 small pieces were collected by sieving the sediments. Unfortunately, most of the facial bones, as well as large part of the cranial base and almost of all the left parietal were not located.

**Figure 1:**
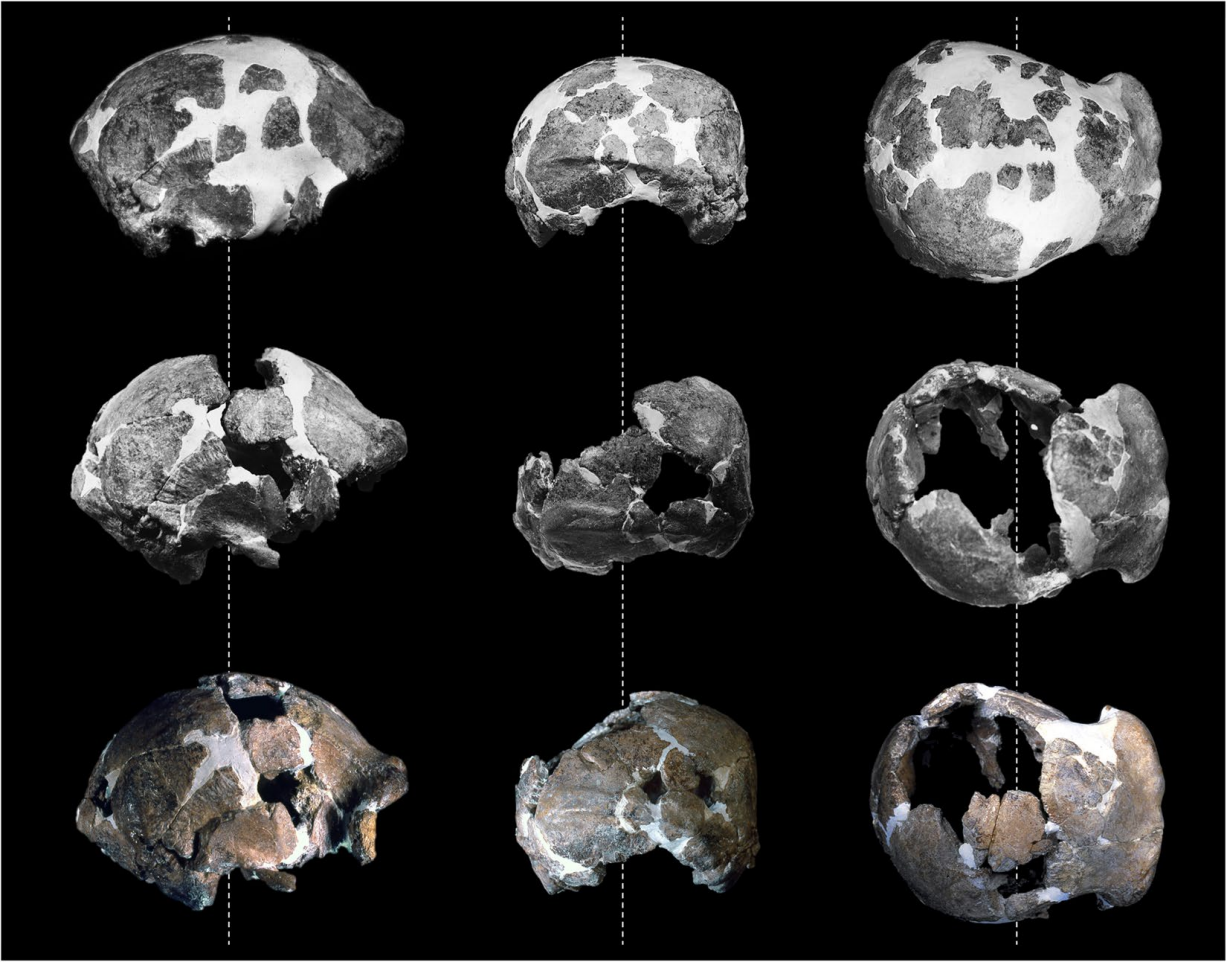
Three stages of the challenging process of restoration of the Ceprano calvarium performed directly on the original specimen by various workers between 1994 and 1999. As described in the text, the reconstruction was originally performed under the direction of the late A. Ascenzi (top row), subsequently corrected by R.J. Clarke (middle), and revised by M.A. de Lumley, F. Mallegni and co-workers (bottom).

Since its discovery, the state of preservation and the incompleteness of the Ceprano calvarium contributed to uncertainty about its taxonomy and phylogenetic position. In several attempts to resolve this problem, the large and anatomically identifiable cranial fragments were submitted to a challenging process of restoration. This took place just after the discovery and was completed in 1999: the final restoration is based on the original reconstruction performed preformed between 1994 and 1996 under the direction of the late prof. A. Ascenzi^2^; this work was subsequently corrected by R.J. Clarke in 1997^17^ and his restoration was further revised by M.A. de Lumley, F. Mallegni and co-workers in 1999^3^ (Fig. 1). The main difference between the first and the subsequent reconstructions involves the removal of many of the original inclusions of dental plaster (i.e. gypsum), as well as the relationship between the anterior and posterior portions of the cranium. On the right side of the calvarium, this relationship now rests on the articulation between the frontal and the parietal bones (along two large segments of the coronal suture), while the greater wing of the sphenoid articulates with the frontal bone and (endocranially) with the temporal squama. On the left side of the calvarium, the temporal bone is largely connected with the preserved part of the sphenoid, which in turn articulates with the frontal bone. The third and current reconstruction^3^ specifically differs from the previous ones for the following improvements: a large fragment of the right parietal (originally considered as a posterior part of the left parietal) has been referred to the anterior corner of the right parietal, joining the frontal bone along the first third of the coronal suture, and was thus repositioned; some small anatomical elements have been added to the restored calvarium, as for example the frontal processes of the zygomatic bones.

Unfortunately, all these reconstructions were made directly with the original fragments and with extensive use of dental plaster, particularly at the end of the first attempt^2^. The subsequent restoration procedures required the removal of great part of the plaster used for the original reconstruction. Medical CT-scans of the calvarium revealed the extent of plaster of at least two types, which pervade the fine texture of the diploic layer of various bones (Fig. S1) and discouraged mechanical removal. The use of chemical solvents were also not possible. Attempts to digitally delete the plaster from the calvarium have also failed, since medical CT scanners do not have enough resolution; therefore, even this procedure did not work either using threshold filters applied globally or working manually on each tomographic slice.

**Figure 2:**
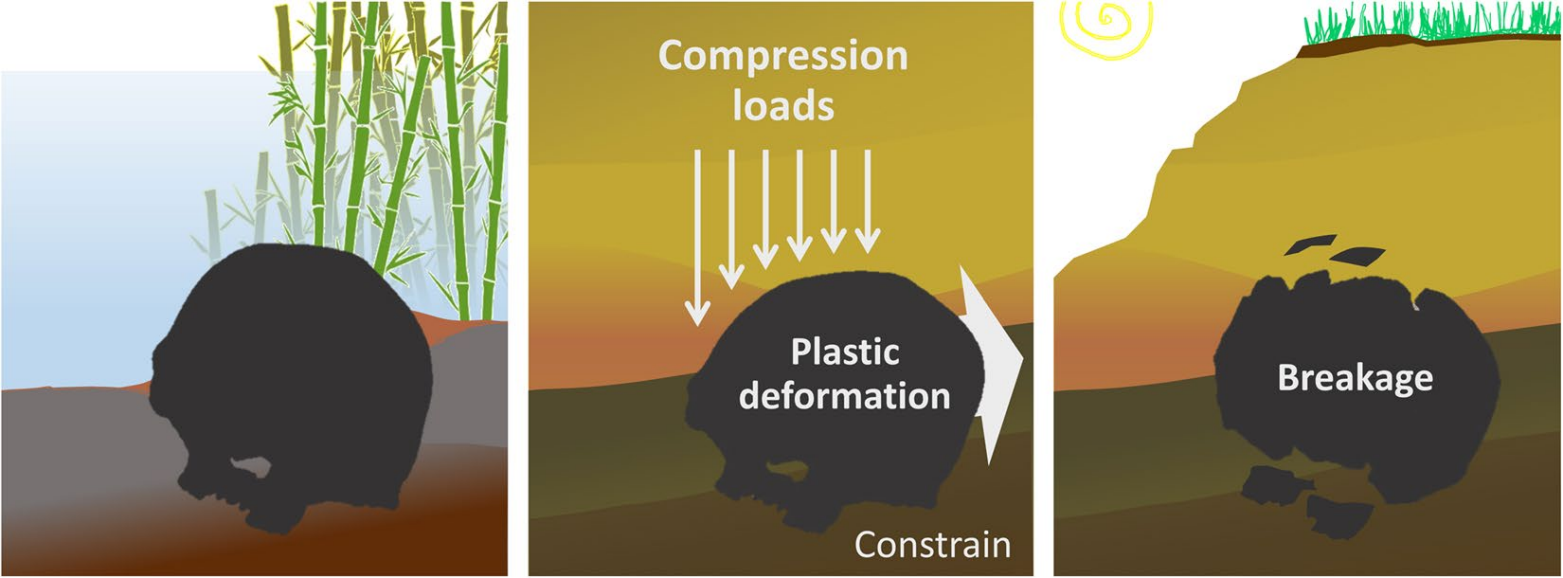
Hypothesized original position of the Ceprano calvarium within the sediments, and subsequent phases of the diagenetic process; plastic deformation occurred before the fossilization of the cranium, with eventual breakage and loss of bony materials on the left and longitudinal fractures on the right.

Nevertheless, it is clear that the Ceprano calvarium requires further restoration efforts in order to be better understood. As a matter of fact, the calvarium appears affected by a deformation involving the postero-lateral vault. The main feature of this deformation is clearly visible in posterior view (Figs 1 and S2), where the cranial vault appears compressed on the left and bent on the right, with comparable but opposite deformation effects^13,18^. This implies an action of taphonomic processes due to the overlying sediments (Fig. 2), which appears to have applied an exertion of a lateral and downward pressure onto the left parietal. This pressure then flattened and fragmented the left parietal, with irregular edges of the preserved fragments and the rest of the bone that became largely missing. By contrast, the right parietal is angulated in coronal sections at the level of the temporal lines, with elongated fractures that run antero-posteriorly along the sub-vertical lateral wall of the cranium. It is probable that the same taphonomic process of deformation affected the occipital squama too, which exhibits an unnatural tilting of the transverse occipital torus leading it to appear clearly sloping toward the right when observed in posterior view (see Figure S2).

Our goal is to examine in detail the effects of these presumed taphonomic events, providing new insight into the original morphology of the specimen. We have chosen to apply methods from virtual anthropology^19^, submitting the calvarium and a number of isolated fragments to X-ray microtomography (μCT). Only with high-resolution 3D imaging we can in fact digitally remove the insertions of dental plaster and separate the fragments. This was a prerequisite for correcting the positions of pieces that were misplaced or misaligned. Eventually, we performed the retrodeformation realignment of the entire volume of the calvarium through a symmetrization procedure in R environment^20–22^. Before the retrodeformation was performed, we followed an analytical protocol that aimed to quantify consistency between the taphonomic hypothesis and the pattern of deformation that affected the calvarium.

## Results and Discussion

**Figure 3:**
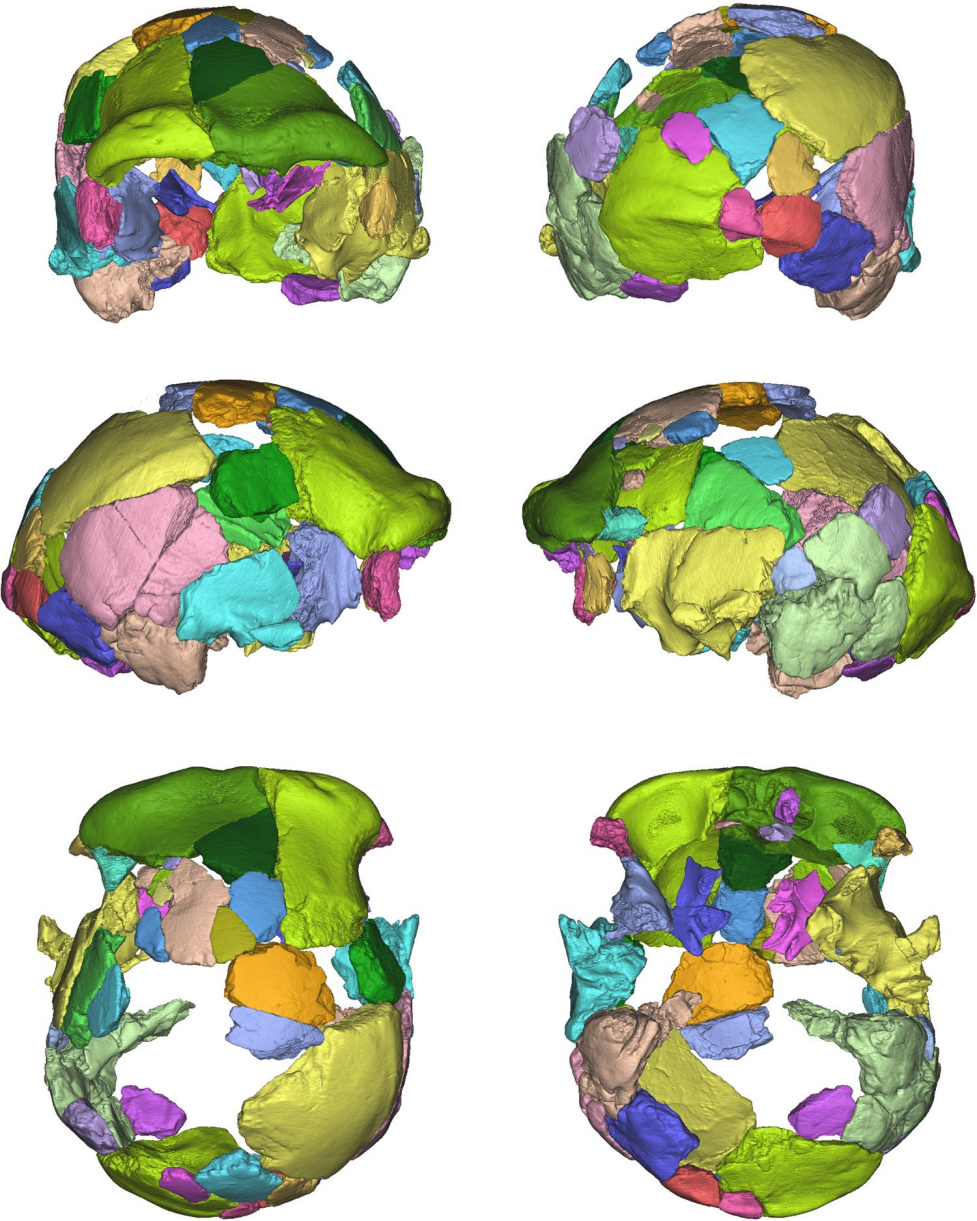
Six canonical views of the segmentation of the Ceprano calvarium after μCT scans, which freed it from the plaster matrix. All the fragments included in the last physical reconstruction^3^ (N = 41) were thus digitally isolated and are distinguished here by color.

Each fossil fragment of the digitized Ceprano calvarium (N = 41) was freed from the plaster matrix and separated – using manual segmentation methods – from the other fragments (Fig. 3). It was not possible to add any of the larger isolated fragments (N = 20; Figure S4) into the new reconstruction; although we were able to determine their anatomical identity (Table S2), none of them displays a clear contact with any of the fragments included in the reconstruction.

**Figure 4:**
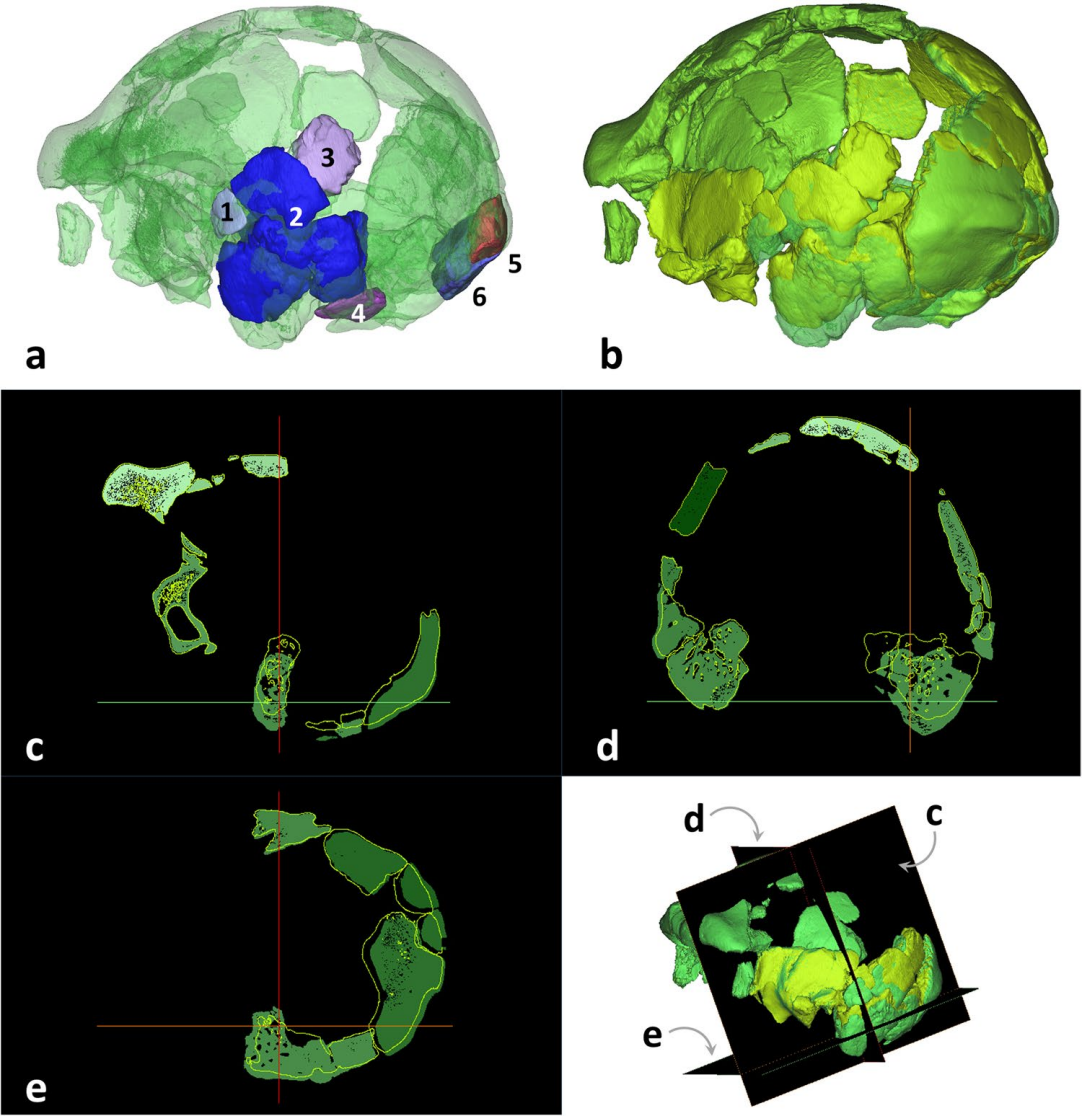
These pictures show changes occurred in our digital reconstruction of the Ceprano calvarium with respect to the last physical one^3^. (**a**) Temporal fragments labeled 1-4 are moved superiorly and rotated anteriorly and connect better with other portions of the left temporal bone; while the occipital fragments 5 and 6 are moved slightly medially to fit better with the occipital bone (compare Figure S6). (**b**) The final result is represented in light green (dark green = former reconstruction). (**c–e**) Three orthogonal sections showing the main differences in the position of the bony fragments 1–4 when moved from the previous reconstruction (solid surfaces) to the newly restored one (empty light contours).

**Figure 5:**
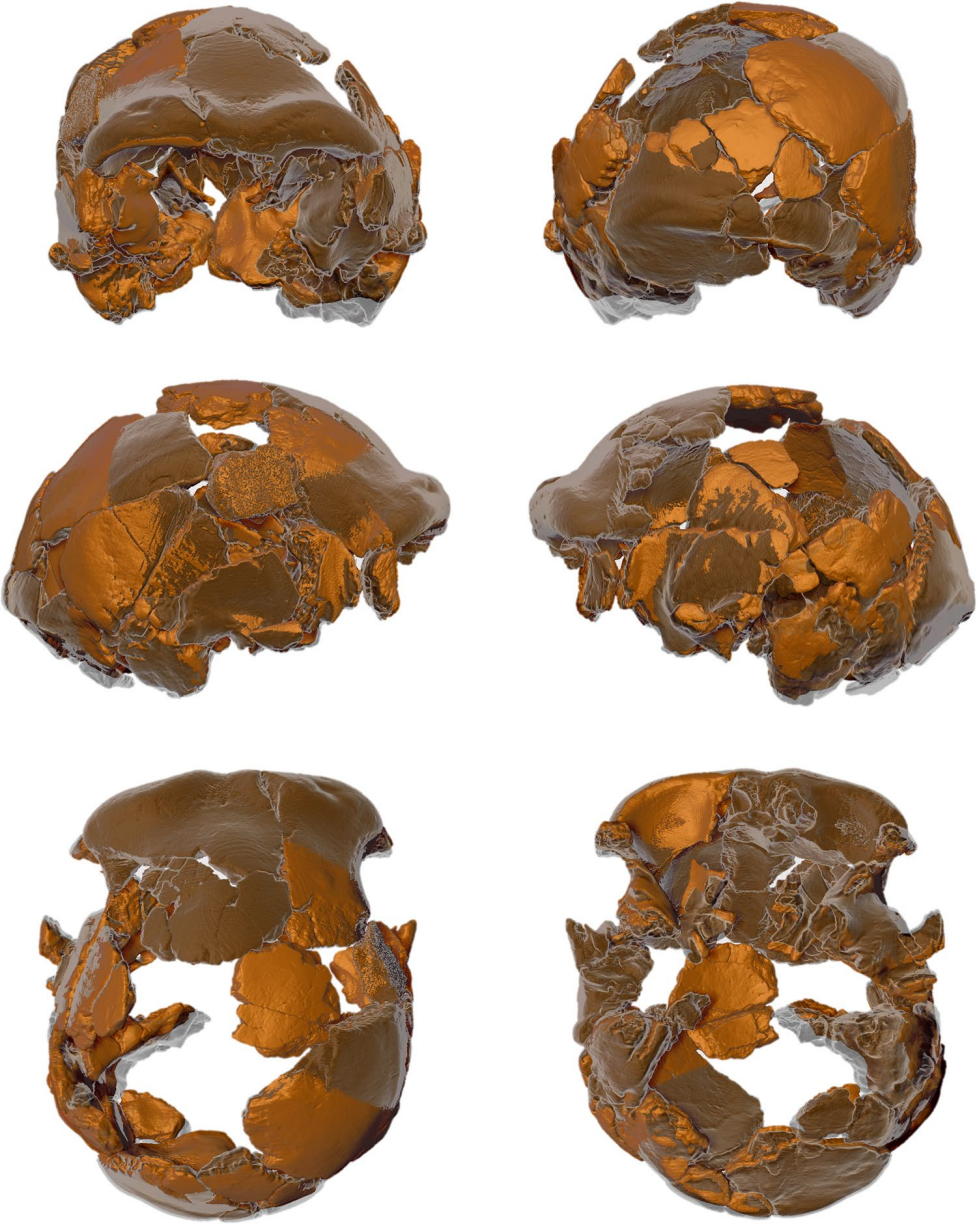
These six views show our digital restoration of the Ceprano calvarium superimposed on the last physical reconstruction. In this representation, transparent (light gray) anatomical parts correspond to previous positions of bony elements that moved to new positions (in light brown), contrasting with those that are unchanged (in dark brown).

When the fossil fragments were freed from the plaster, it was clear that the original reconstruction needed further intervention. It became obvious that several of the fragments had been misplaced or misaligned. We were able to implement a series of corrections to the relative position of each digital fragment. The consequences of these changes are visible in Figs 4–5 (compare with Figure S5).

Specifically, the temporo-parietal region of the left side of the calvarium required considerable adjustment. The right temporal bone had been successfully reconstructed by previous research efforts, whereas the left temporal bone lacked bony contacts anteriorly (for example, at the base of the zygomatic arch) and posteriorly (where an ossicle at asterion is missing). Thus, looking at the first physical reconstruction^2^, this region appears projected posteriorly and laterally with respect to the midsagittal plane (Figs 1 and S2). This displacement was never corrected^3,17^ and confers to the left side of the calvarium an unnaturally irregular profile, as compared to the right side from a superior view. At the same time, the left mastoid complex appears displaced inferiorly, when compared in posterior view to the mastoid of the right side.

Our digital repositioning of these skeletal elements started by moving the fragments labelled 1–4 superiorly and anteriorly (see Fig. 4), in order to find a better fit with the anterior portion of the left temporal bone. Specifically, fragment 1 was isolated and then realigned to firmly connect to fragment 2 (posterior portion of the left temporal bone). To accomplish this, we used the relationships between the various anatomical elements that are visible on the right side to guide us. These elements included the relative position of the angular torus, the inferior temporal line and a terminal segment of the supramastoid crest. Because of this effort, the segment of the temporal line that is visible on fragment 1 now rests in a natural continuity with the temporal line preserved on the anterior portion of the temporal bone (parietal component of fragment 2).

The new position of fragment 1 produced several cascading effects, starting from a hiatus of a few millimeters that was filled by moving fragment 2 superiorly and rotating it medially. In addition, the anterior portion of the temporal bone was rotated medially, in order to fit with the new position of the mastoid complex (Fig. 4). These movements reduced the lateral projection of the left parietal profile, while the preserved portion of the sphenoid on the left now better approximates the level of the same region preserved on the right side (compare Fig. 5). Eventually, given the adjusted anterior position of the left temporal bone, the occipital bone was also repositioned, followed by the entire right side of the cranial vault, which shifted posteriorly and slightly superiorly.

Another important repositioning involved the new orientation of fragments of the occipital squama labeled 5 and 6 (see Fig. 4). These have been placed more medially than in the previous reconstruction; in particular, a small fragment specific to the inion landmark (fragment 5 in Fig. 4), including a portion of the external occipital protuberance, was rotated towards the nuchal plane (see Figure S5). As a result of this repositioning, the residual part of the external occipital protuberance now makes direct contact with a cranial fragment housing the external occipital crista.

After digital restoration, the taphonomic deformation that affected the specimen required an extensive study of the deformation pattern. This assessment mainly involved the parietal bones (with crushing of the left parietal and bending of the right parietal) and the occipital bone (including the tilted transverse torus). This evaluation was carried out using a protocol that combined Thin Plate Spline (TPS) and Finite Element Analysis (FEA) approaches, as described in the “Material and Methods” section of this paper.

**Figure 6:**
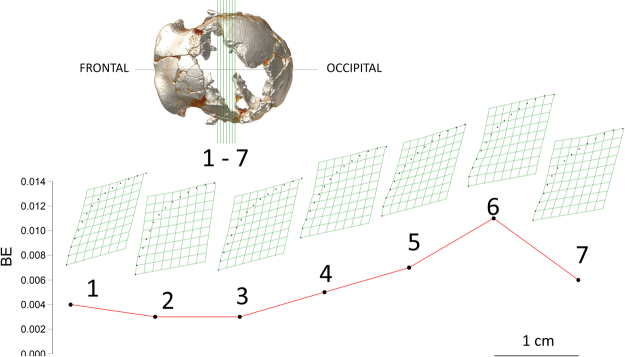
Transformation grids and associated bending energy values obtained after superimposition of the left/right profiles of the external surface of the parietal walls along seven coronal cross sections, equally spaced 1 cm apart.

The TPS analysis (Fig. 6) showed that the recorded values of Bending Energy (BE) associated with left/right asymmetry of the cranium are close to zero, displaying similar values along all the portions of cranial vault examined (Fig. 6 and Table S3). This result is expected for a deformation that follows an affine or uniform pattern of warping^23,24^. It is also in accordance with the hypothesis put forward by Bruner and Manzi^13^, which implies a constant pressure exerted by the sediment pack on the bony material during early phases of the diagenesis, when the bones were still rich in collagen. According to this hypothesis, the pressure exerted on the left parietal was responsible for its crushing, on the one hand, and for the symmetrical deformation of the right parietal, on the other hand, while the entire bi-parietal vault was uniformly translated from left to right. This is also highlighted by the considerable bending of the right side of the cranium at the level of the temporal lines, associated with the antero-posterior fractures of the lateral wall. At the same time, the unnatural inclination toward the right side of the transverse occipital torus is consistent with this process of deformation.

Given the results of the TPS-based analysis, we modeled with FEA the plastic response of a cranium submitted to a single event of deformation. This is reasonable since, a human cranium, like other complex skeletal parts^25,26^, rarely tolerates more than a single episode of plastic deformation. The results of the FEA analysis (Figure S6) shows that the deformation induced on the model (the Middle Pleistocene cranium from Petralona, Greece) is fully consistent with what is visible on the Ceprano calvarium. When observed in posterior view, we note both in the model and in Ceprano a rather flattened left parietal and a more vertical right wall of the cranium, which increases at the level of the temporal lines. Moreover, there is a consistent clockwise tilting of the occipital squama, which is reflected by the inclination of the internal occipital crista.

On the basis of these results, we were then able to apply the retrodeformation algorithm^22^ to our reconstruction of the calvarium. It is worth noting that the retrodeformation is effective and reliable here, because we are dealing with a simple, affine deformation (where the deformation of one side is likely to be the opposite of the deformation on the other side). Thus, the retrodeformed model occupies a point in shape space that is likely to be close to that occupied by the original. More complex deformations are more difficult to restore based on asymmetry, for the simple reason that the deformations themselves are unlikely to be opposite and equal between sides, despite the algorithm we used^22^ has been developed in order to work also in case of non-affine deformations.

**Figure 7:**
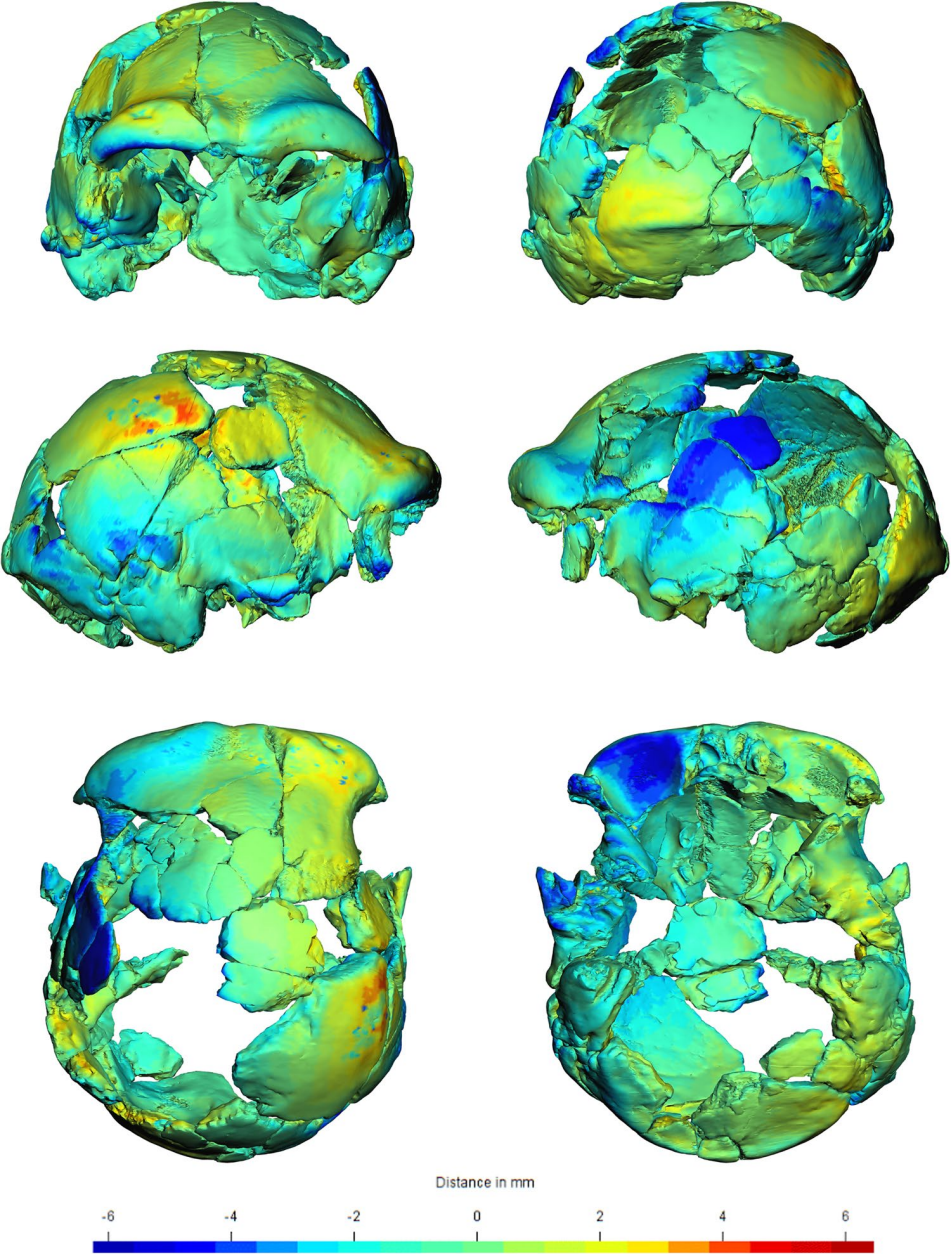
The retrodeformed Ceprano calvarium. Differences from our digital reconstruction (reported in Fig. 5) are expressed by colors (ranging from −6 to 6 mm). These colors represent inter mesh distances; moving toward the yellow-red extreme of the spectrum we find the regions of the new reconstruction that are pushed inward and are now less angulated with respect to the non-deformed reconstruction; vice versa for the blue areas.

The result is a model of the Ceprano calvarium that we consider as close as possible to the original morphology of the specimen before the taphonomic deformation occurred. In order to quantify and visualize this result, the retrodeformed shape of Ceprano is represented as mesh distances (ranging from −6 to + 6 mm) from the shape of our restoration of the specimen (Fig. 7).

## Conclusions

The main issues resolved by our digital assessment of the Ceprano calvarium are as follows: 1) realignment of the parietal bones providing the original profile of the parietal walls of the cranium; 2) rotation of the occipital transverse torus and the adjacent portions of other occipital bone fragments; 3) symmetrisation of the occipital-temporal regions relative to the zygomatic and mastoid complexes; 4) realignment of the spheno-ethmoidal regions; 5) moderate symmetrisation of the frontal squama.

These results indicate that the protocol we adopted has provided a new and more reliable shape of the Ceprano calvarium, one that better approximates the original form of such an important specimen of the Middle Pleistocene^1^. We may thus assume that the realigned morphology corresponds to the shape of the calvarium before the overlying sediments compressed the cranial vault. Yet, no morphometric changes took place compared to previously published morphometric data^3^, because standard anthropological measures do not register variations in the regions that were mostly distorted.

Given the deformation pattern, it should have occurred when the cranium was still rich in collagen. As a result, the plastic deformation reached a threshold of breakage and produced two main effects: a severe fragmentation of the left parietal and the occurrence of the antero-posteriorly elongated fractures that are visible on the right parietal. Consequently, the compressive loads exerted on the left side were transmitted to the other side of the cranial vault, causing the unnaturally angled right parietal wall along with several other specific defects, such as the oblique transverse occipital torus; the frontal bone, by contrast, does not seem to have been significantly affected by the postmortem deformation process.

The retrodeformed shape we obtained reduces (at least in intensity) some of the peculiarity of the Ceprano calvarium that have been claimed. For instance, when Mallegni and colleagues^15^ named the new species *H. cepranensis*, they based their tentative cladistic analysis and conclusions on a single apomorphy (the relative short length of the calvarium) that should distinguish a cluster including only Ceprano and the African calvarium BOU-VP-2/66, referred to as Daka^27–29^. On the contrary, Ceprano shares a number of shape specific features with most of the crania that are commonly included in the hypodigm of *H. heidelbergensis*. After the present reconstruction and retrodeformation (see Fig. 7), both the angulation of the right parietal wall and the depression of its left side have disappeared. Thus, as a result of the realignment of cranial fragments, the shape of the Ceprano calvarium appears to be closer to Middle Pleistocene specimens from Africa and Eurasia, such as Kabwe and Petralona (see Figure S8). This conclusion places more firmly the Italian specimen among a group of fossils that represents the species that was likely ancestral to Neanderthals, modern humans and the so-called “Denisovans”^1,8,12^.

Another implication of our work helps to better locate the Ceprano calvarium chronologically, with respect to its depositional history. For almost a decade after discovery, the specimen was dated to the end of the Early Pleistocene (800–900 ka)^2,3^ More recently, it has been dated to the interval between 430 and 385 ka; this was done using multidisciplinary evidence collected during systematic excavations of the last decade^4^. Moreover, this chronology is consistent both with the geomagnetic pattern recorded in the area^30^ and with the Ar/Ar date of 353 ± 4 ka^31^ obtained from K-feldspars that were sampled above the gray clay layer in which the cranium was located.

It should be pointed out, however, that the original late Early Pleistocene age of the Ceprano fossil is still maintained by some workers^32^. This claim is based on the assumption that the calvarium was discovered in secondary deposition within younger sediments^32^.

Conversely, our analysis support the idea that the calvarium was located in a primary deposition. The rationale for this conclusion relies on the fact that the taphonomic process of plastic deformation and final fragmentation of the specimen occurred when the cranial bones were still rich in collagen, that is before mineralization took place. It is improbable that all the cranial fragments moved as a whole during geological time from their primary deposition^33^; while, vice versa, the fact that all these fragments were found in one single location supports a primary deposition of the fossil specimen.

A “fresh” (i.e., not yet “fossil”) bone can be plastically deformed before it breaks because it is still rich in collagen and because the calcium crystals that constitute large part of the bony matrix are not yet substituted by other minerals, as happens during the process of mineralization^34^. Such a diagenetic process may occur in environments that are rich in water, like a riverbed or a perilacustrine paleosol; the latter was probably the case in the Ceprano area^5^. This depositional environment is consistent, in turn, with the absence of taphonomic signatures on the calvarium, which lacks any gnaw-marks, weathering signatures (such as radial flaking) or transport-induced abrasions, indicating that it was not affected by long exposure and/or fluvial reworking^5^. Further, the normal processes of raising and lowering of water levels – demonstrated by pedofeatures of the sediment in which the cranium was included^5^ – likely dispersed other bony elements of the same skeleton and rapidly added sediments on the cranium.

Therefore, the combined evidence collected here supports the hypothesis that, shortly after death, the Ceprano calvarium was permanently buried in the perilacustrine environment where it was found.

## Material and Methods

### Microtomography and digital segmentation

The Ceprano calvarium was digitally recorded with the μCT system available at the Multidisciplinary Laboratory of the “Abdus Salam” International Centre for Theoretical Physics in Trieste, Italy^35^. Due to the size of the specimen, it was scanned in three partially overlapping parts, by changing the vertical spatial coordinate. The X-ray μCT acquisitions were carried out with a source voltage of 145 kV, a current of 206 µA, a 3 mm aluminium filter, an exposure time of 1 s and recording 4800 projections of the sample over 360°. The resulting μCT slices, reconstructed using the commercial software DigiXCT in 16-bit format with a pixel size of 42.41 µm, were merged in order to obtain a single 3D volume rendering of the whole specimen. In addition, twenty isolated bony fragments (see Figure S4) were separately scanned using the same analytical parameters.

### Modelling the taphonomic deformation

In order to quantify the pattern of deformation that occurred in the Ceprano cranium during the taphonomic process, we followed a protocol that combines Thin Plate Splines (TPS) and Finite Element Analysis (FEA).

TPS is an interpolator for one configuration of landmarks onto another^36,37^. It minimizes the integral of the squared second derivatives, a quantity referred to as bending energy (BE), which measures the amount of local shape deformation or landmark displacement^38^. In affine transformations, which correspond to a tilting without bending, the bending energy is equal to zero, while it increases in magnitude when bending (non-affine transformations) occurs.

We traced the coronal profile of the parietals following the same rationale reported in Bruner & Manzi^13^, who used a single digital section approaching the maximum width of the cranium. Thus, after the interpolation of the missing fractions into a reasonable minimally bent profile, we outlined the right and left parietal external profiles. We repeated the sampling for seven coronal sections, evenly spaced by 1 cm and for a total length of 6 cm along the cranial walls (see Fig. 6). These profiles were reduced to a 30 semilandmarks configuration and superimposed (after mirror imaging) using the Procrustes procedure. We then used the BE values as a measure of the total warping that affected the calvarium during the taphonomic process.

The aim of the TPS-based procedure was to assess whether an affine or non-affine transformation was most likely to have occurred during the taphonomic process. We were then able to apply FEA, simulating either a single or multiple vector(s) of compressive loadings. FEA is a powerful method of mechanical simulation, which may be applied to the dynamic behavior of a given physical object; it acts by reducing a complex geometry into a finite number of elements with simple geometries connected by shared nodes^39–43^. It can be used to investigate and predict deformations produced by geological forces acting on vertebrate fossils during diagenesis^26^.

Eventually, a 3D model based on the Middle Pleistocene cranium from Petralona was generated with Amira 5.4.5 and imported into VoxFE Ver. 1.0^44^. In order to simulate the original loading conditions, the model was constrained on the cranial base at the level of the occipital condyles, with the compressive loadings acting on the whole surface of the left parietal bone. The model was simplified and allocated homogeneous material properties of compact bone (Poisson’s ratio = 0.3, and Young’s modulus = 16.9 GPa). It was solved using 50000 iterations and a tolerance = 1e-09.

### Retrodeformation

Retrodeformation of the Ceprano calvarium was based on a set of bilateral cranial landmarks. Our aim was to remove postmortem taphonomic damage to the cranium shape. The traditional approaches commonly used to symmetrize a 3D model are based on the definition and acquisition of anatomical points (e.g. ref.^45^). Unfortunately, anatomical landmarks are most often not uniformly distributed, and in some portions are completely absent. The protocol recently developed by Schlager^22^ overcomes this problem. Besides the traditional methods, this procedure uses bilateral landmark sets and symmetrical semilandmark sets. In doing so, the configuration of points adequately represents the entire geometry of the structure. In detail, this protocol^22^ is an implementation of the closed form solution proposed by Ghosh and colleagues^46^, improving the placement of the semilandmarks with respect to other methodologies^47,48^ and adding a symmetrisation step to make these sets of points geometrically homologous^22^.

The retrodeformation of Ceprano required the acquisition of 18 bilateral landmarks and 4 semilandmark patches, for a total of 172 points (Figure S3). Subsequently, we applied the function *symmetrize, relaxLM*, and *retroDeformMesh* from the Morpho R package^21,22^.

One of the main problems we faced in positioning the semi-landmarks bilaterally on the external surface of Ceprano is that the left parietal is largely missing. Thus, we reconstructed the deficient portions using a CAD protocol in order to draw a number of best fitting curves of the original profile of the lateral wall, based on the preserved portions. Specifically, we used a network of a 20 polynomial (Bèzier) curves that were built connecting three or more points aligned on a given vector along the preserved portions of bone^49^, according to the definition of the Bèzier curves^50^. Bèzier curves can be defined from a set of Cartesian coordinates in 2 or 3 dimensions obtaining a parametric curve interpolation in a plane or in a three-dimensional space^47,51^. Thus, it is possible to acquire a series of control points on the actual surface of a 3D model and calculate the Bèzier curves that cross its missing portion. It is then possible to obtain a series of landmark points evenly spaced along the curves. Therefore, using Mimics Materialise 17.0, we calculated 20 sets of evenly spaced points starting and ending on the preserved region of the Ceprano calvarium and crossing the missing portions of the left parieto-temporal wall. The cloud of points was eventually used to create a digital approximation of the external surface of the braincase applying a Delaunay algorithm^52^ in Amira 5.4.5 (Figure S3).

### Data Availability

All data are available upon request.

## Electronic supplementary material

Supplementary info
