# Supplementary material for: Digital reconstruction of the Ceprano calvarium (Italy), and implications for its interpretation
Source: Sci Rep. 2017 Oct 25;7:13974. doi: 10.1038/s41598-017-14437-2 (PMC5656598; doi:10.1038/s41598-017-14437-2)
Supplement: Supplementary file 1 — Supplementary info [file 41598_2017_14437_MOESM1_ESM.pdf]

Supplementary Information

Digital reconstruction of the Ceprano calvarium (Italy), and implications for its interpretation

Fabio Di Vincenzo [1, 2], Antonio Profico [1, 2], Federico Bernardini [3, 4], Vittorio Cerroni [5], Diego Dreossi [6], Stefan Schlager [7], Paola Zaio [5], Stefano Benazzi [8, 9], Italo Biddittu [2], Mauro Rubini [2, 5, 10], Claudio Tuniz [4, 3, 11], Giorgio Manzi [1, 2, *]

1. *Dipartimento di Biologia Ambientale, Sapienza Università di Roma (Italy)*
2. *Istituto Italiano di Paleontologia Umana, Roma (Italy)*
3. *Centro Fermi - Museo Storico della Fisica e Centro di Studi e Ricerche 'Enrico Fermi', Roma (Italy)*
4. *The 'Abdus Salam' International Centre for Theoretical Physics, Trieste (Italy)*
5. *Italian Ministry of Culture, Anthropological Service, Roma (Italy)*
6. *Electra - Sincrotrone Trieste, Trieste (Italy)*
7. *Department Biological Anthropology, University Medical Center, Freiburg (Germany)*
8. *Department of Cultural Heritage, University of Bologna (Italy)*
9. *Department of Human Evolution, Max Planck Institute for Evolutionary Anthropology, Leipzig (Germany)*
10. *Dipartimento di Archeologia, Università di Foggia (Italy)*
11. *Centre for Archaeological Science, University of Wollongong (Australia)*

* Corresponding author

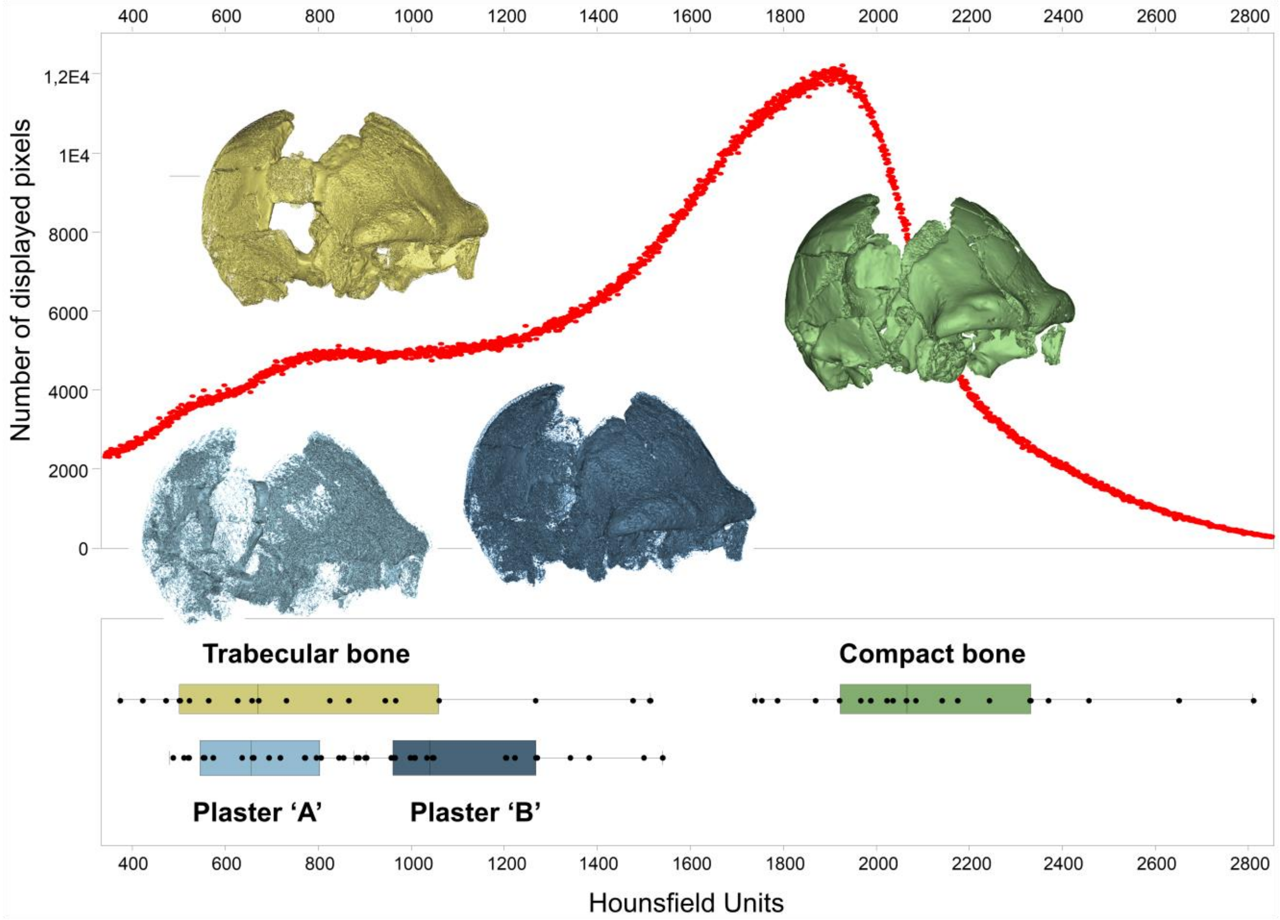

Figure S1. The bimodal distribution of the attenuation curve associated with the medical CT-scan of the cranium (top) do not properly discriminate plaster from the fossil material. The Hounsfield ranges associated with the fossil bone and the plaster widely overlap. Actually, direct measurements performed with Mimics 11.0 – which are reported here as box plots related to the different materials (compare Table S1) – show that at least two different types of plaster are present, probably corresponding to the reconstructions of 1995 and 1999 respectively. Both fall within the range of attenuation of the mineralized trabecular component of the cranial bones; at this resolution level, they cannot be removed neither automatically (using a series of threshold filters) nor by manual segmentation.

	**MIN**	**MAX**	**S.D.**
**Trabecular bone**	332.9	1497.2	378.8
**Compact bone**	1758.0	2845.7	293.6
**Plaster 'A'**	471.7	890.1	138.1
**Plaster 'B'**	863.5	1520	197.1

Table S1. Range of Hounsfield values for bone and plaster respectively

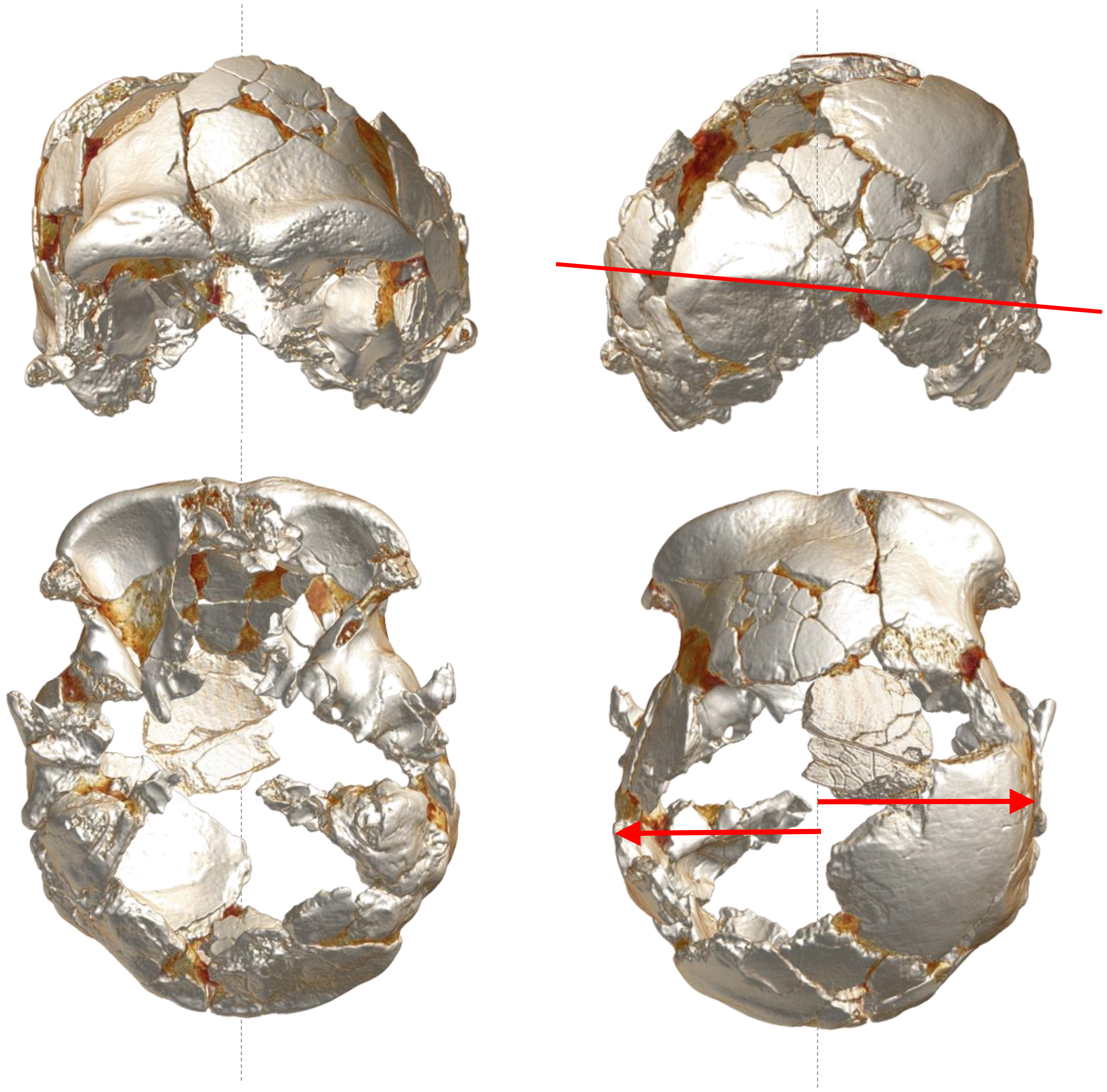

Figure S2. The calvarium is affected by diagenetic deformation of the postero-lateral vault. The deformation of the calvarium is due to diagenetic processes and is not related with the frangmentation that occurred during the accidental circumstances of its recovery or with biases in the reconstruction. The frontal squama and supraorbital torus appear largely undistorted in respect to mid-sagittal plane. In posterior view it is well evident that the transverse occipital torus resulted tilted and that the left parietal being flattened and largely fragmented/destroyed), while the controlateral wall was unnaturally warped being more angulated, with elongated fractures. In ventral and superior view the maximum lateral projection in the left side is unnaturally shifted backward (red arrows).

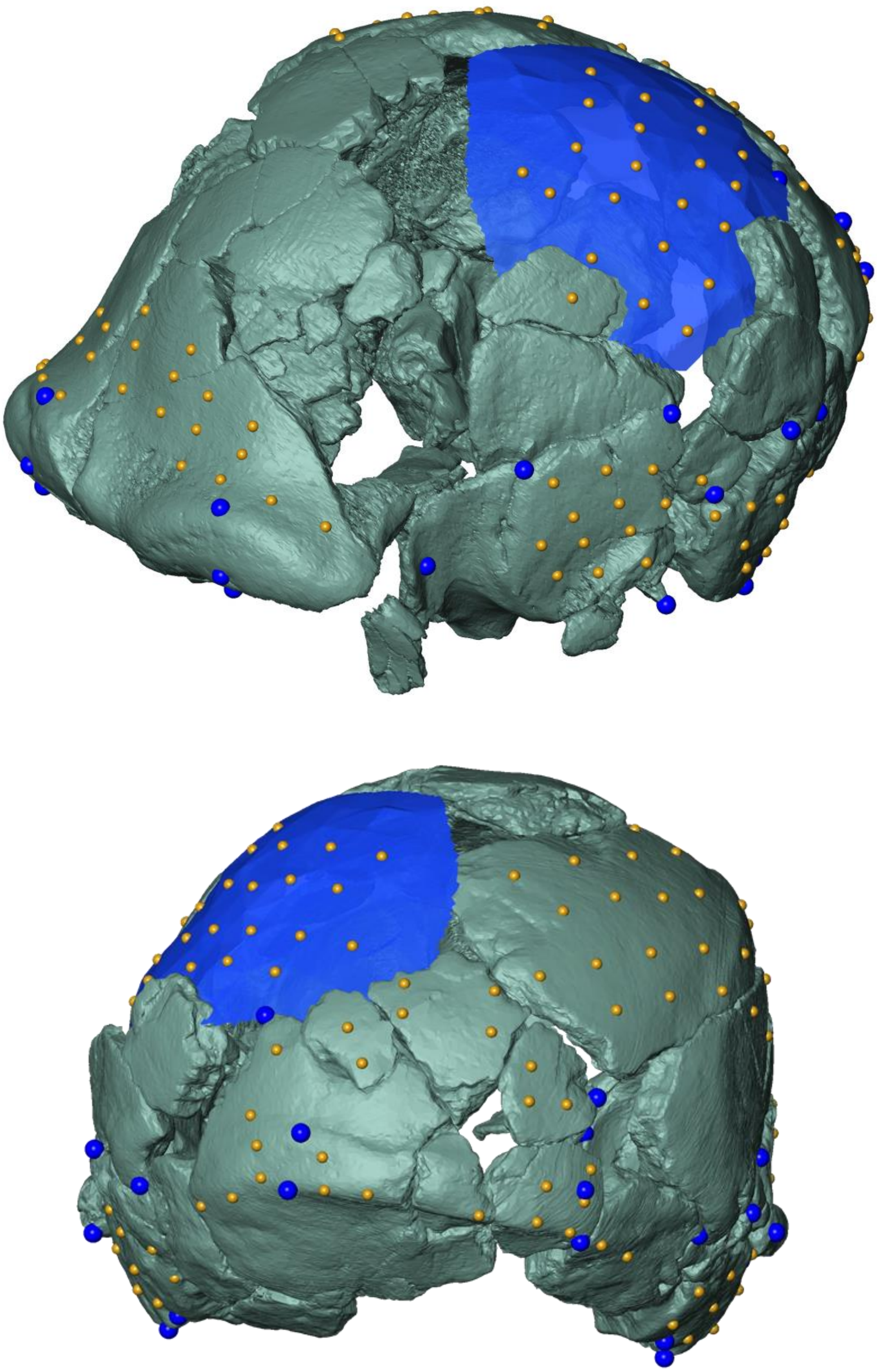

Figure S3. The symmetrical configuration of landmarks (blue spheres) and semi-landmarks (yellow spheres) used to obtain the retrodeformation of the Ceprano calvarium to its alleged original morphology. A virtual surface (showed in transparence on the calvarium) simulating the curvature of the missing left parietal wall was obtained using a network of Bezier's curves starting from the preserved portion of the occipital, the left temporal and the right parietal bones. This surface was created in order to place on it the corresponding set of semilandmarks that were already placed on the right parietal.

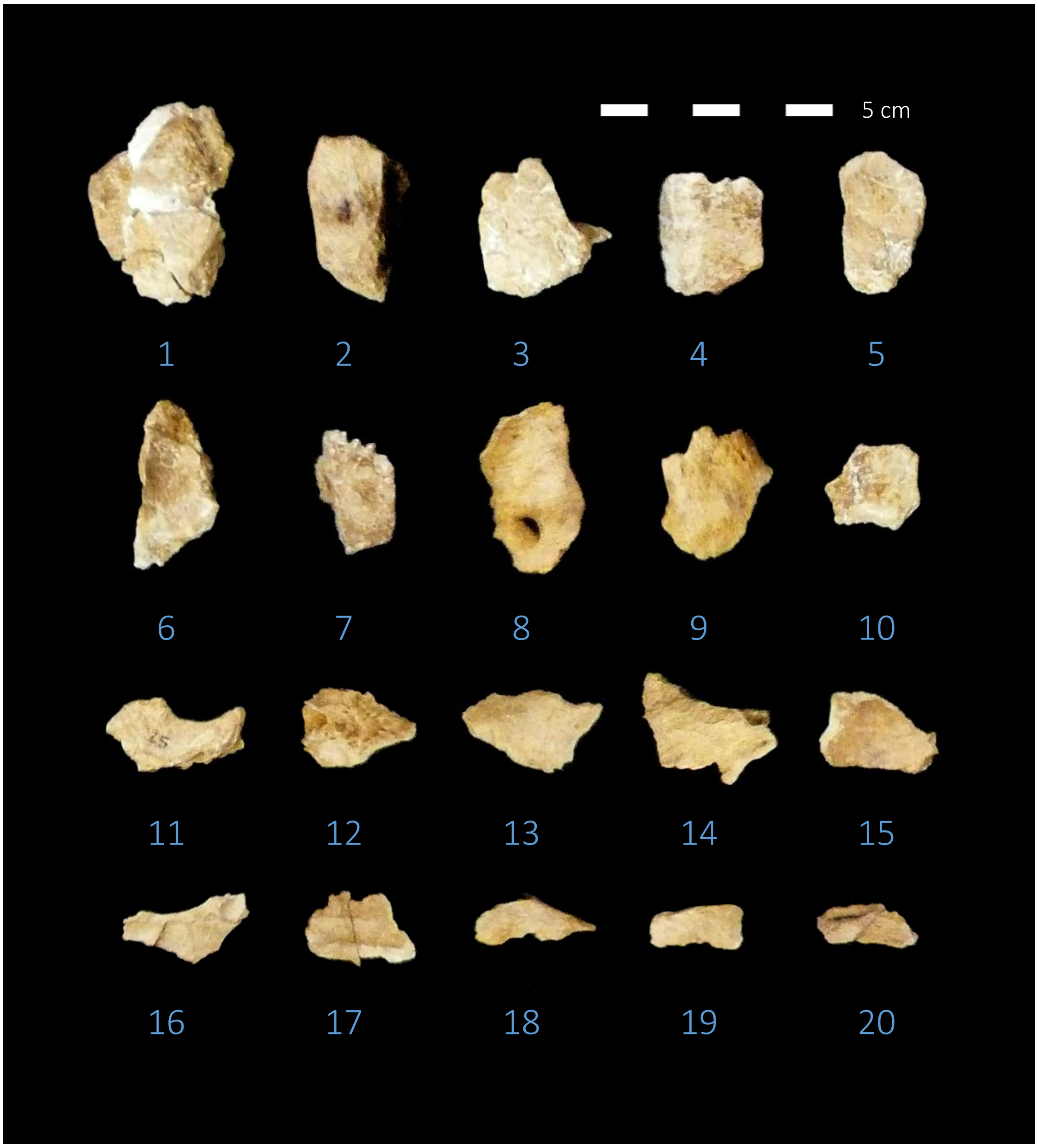

Figure S4 After the first recovery of about 50 large fragments belonging to the same cranium, additional more minutes fragments for a total of about 200, were collected by sieving the sediments from the original layer, soon after the discovery. These "minor" fragments were discarded by the reconstruction process even if 25 of them (figured here) have a size greater than 20 mm and their anatomical original position can be identified. In particular, 8 fragments, including 3 with preserved sutural denticles, belong to parietal-occipital vault, 4 are part of the wall of the orbit, 1 refers to the right side of the occipital bone with preserved the jugular tubercle and part of the hypoglossal canal, 1 is related to the right portion of the sphenoid and preserves the foramen ovale, 4 other fragments are also referable to the sphenoid and 2 remain of dubious attribution. Nevertheless, although we were able to identify their anatomical identity, these fragments were not added to the new reconstruction because the lacking of contact surfaces with other fragments already positioned in the reconstruction.

Table S2. The fragments of the calvarium not placed in the reconstruction. For each fragment the more probable osteological identification and position is reported.

LABEL	DESCRIPTION
Fragment 1	Fragment of the vault (parietal or occipital) with most of the endocranial surface preserved<br>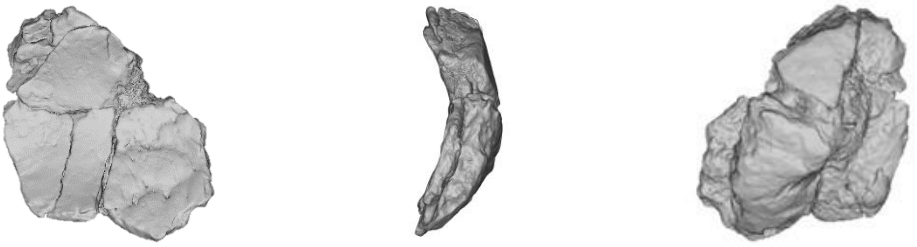
Fragment 2	Fragment of the vault (occipital ?)<br>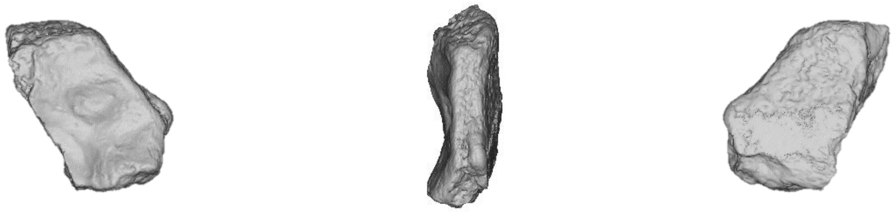
Fragment 3	Fragment of the vault (parietal ?)<br>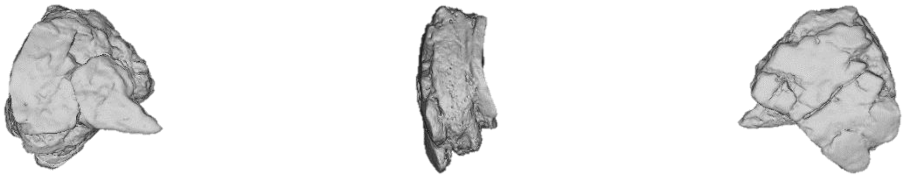
Fragment 4	Fragment of the vault (parietal ?) with preserved some sutural denticles<br>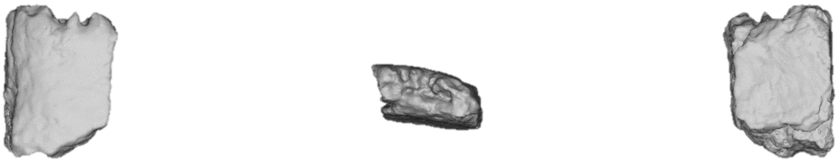
Fragment 5	Fragment of the vault, probably the parietal with only preserved the endocranial surface with some vascular impressions<br>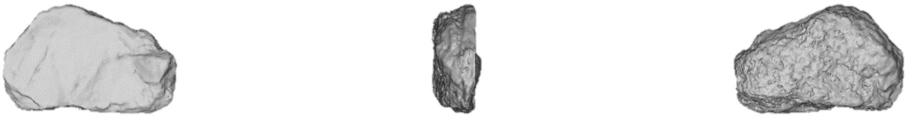

Fragment 6	Fragment indet. (occipital ??) with only preserved the endocranial surface
	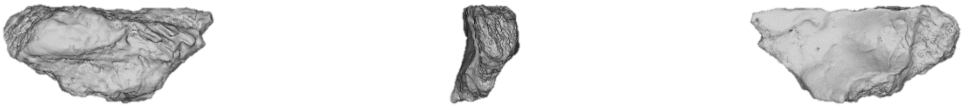
Fragment 7	Fragment of the vault (probably parietal) with preserved some sutural denticles (probably of the coronal suture)
	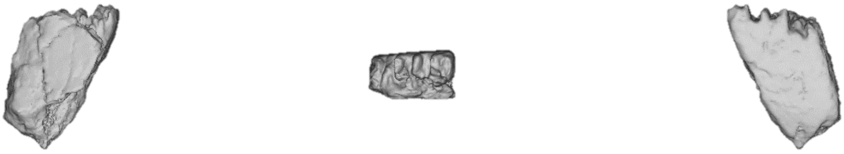
Fragment 8	Portion of the right lateral part of the occipital bone with preserved the internal (endocranial) surface with the jugular tubercle and part of the hypoglossal canal, the fragment appears transversally cut just below the base of the condyle (not preserved). This fragment have not the left counterpart preserved
	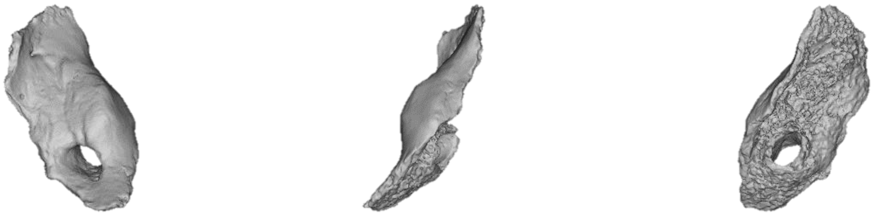
Fragment 9	Fragment indet. largely pneumatized
	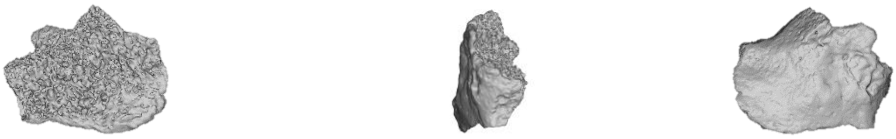
Fragment 10	Fragment of the vault (probably parietal) with some sutural denticles preserved
	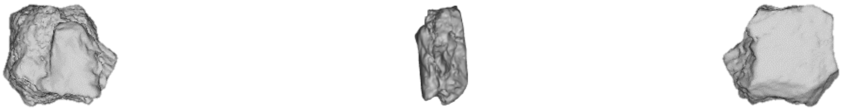

Fragment 11	Fragment indet. (sphenoid ?? temporal ??)<br>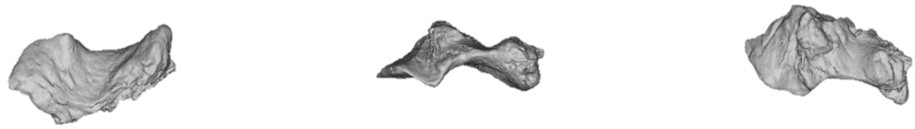
Fragment 12	Fragment indet. (sphenoid ?) with preserved on the surface traces of vascular impressions and foramina (??)<br>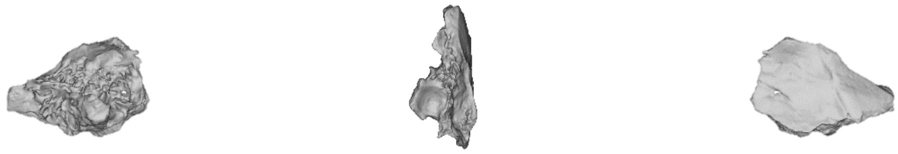
Fragment 13	Fragment indet. (sphenoid ?) with preserved a foramen (?)<br>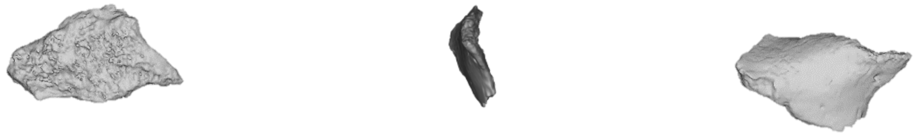
Fragment 14	Fragment indet.<br>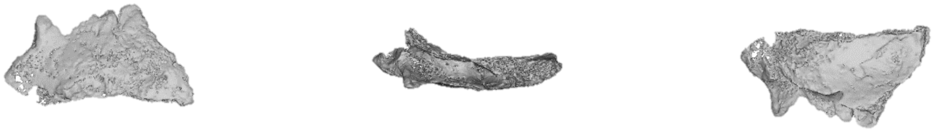
Fragment 15	Fragment indet. (frontal, roof of the orbit ?) with the presence of small pits (?)<br>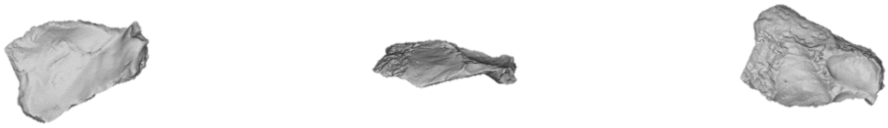

Fragment 16	Fragment indet. (frontal, roof of the orbit ?) with a foramen (?) . SI ARTICOLA CON IL FRAMMENTO 20 ??<br>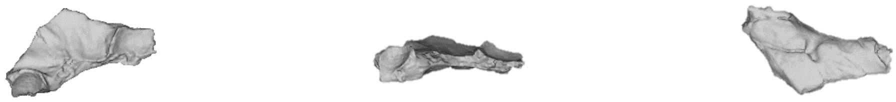
Fragment 17	Fragment indet. (frontal, roof of the orbit ?) . SI ARTICOLA CON I FRAMMENTI 20 E 21 ??<br>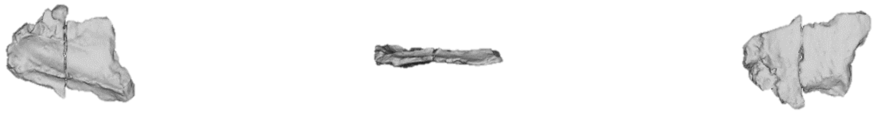
Fragment 18	Spine of the wing of the sphenoid ?<br>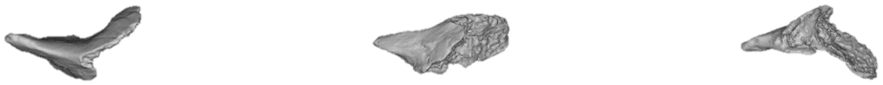
Fragment 19	Fragment indet. (frontal ?) With preserved foramina<br>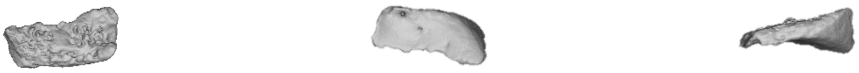
Fragment 20	Portion of the right posterior part of the sphenoid bone preserving the foram ovale. This is a symmetrical fragment; the left foramen ovale is preserved even if the left GWS is affected by a large pathology (Ascenzi, Benvenuti & Segre, 1997)<br>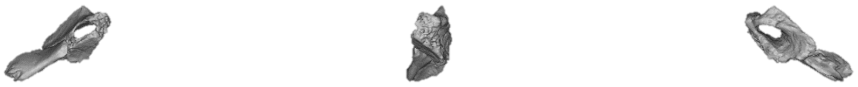

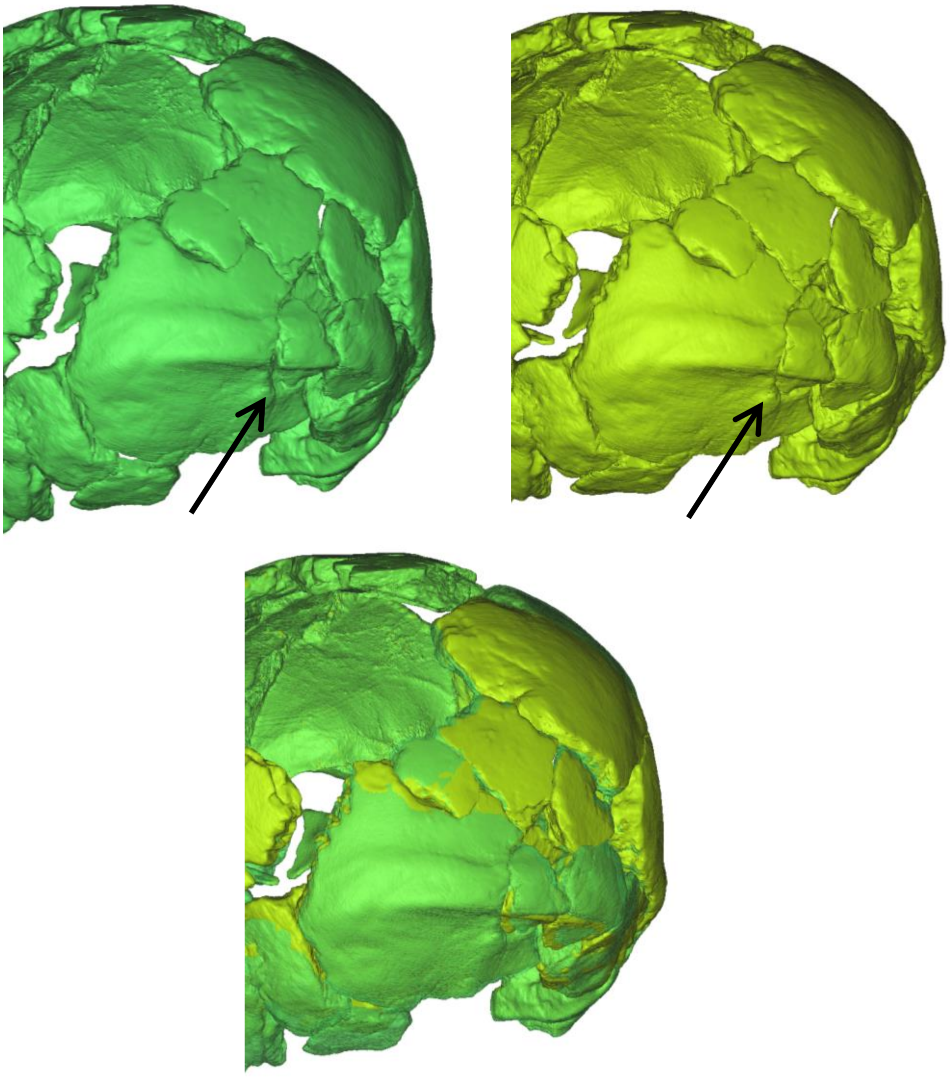

Figure S5 In the former reconstruction the contact between the preserved portion of the external occipital crista and the occipital protuberance appear broken and shifted transversally. The occipital protuberance is largely preserved on a small fragment that in the new reconstruction was moved backward and inferiorly to take direct contact with the occipital crista (black arrow). On the bottom the two reconstructions superimposed.

Table S3. The values of the bending Energy recorded superimposing the profile of the left parietal wall on the right side and vice versa along the coronal sections indicated in Figure 6.

**SECTION LEVEL**	BE SX > DX	BE DX > SX
1	0.010	0.004
2	0.007	0.003
3	0.009	0.003
4	0.015	0.005
5	0.023	0.007
6	0.031	0.011
7	0.019	0.006

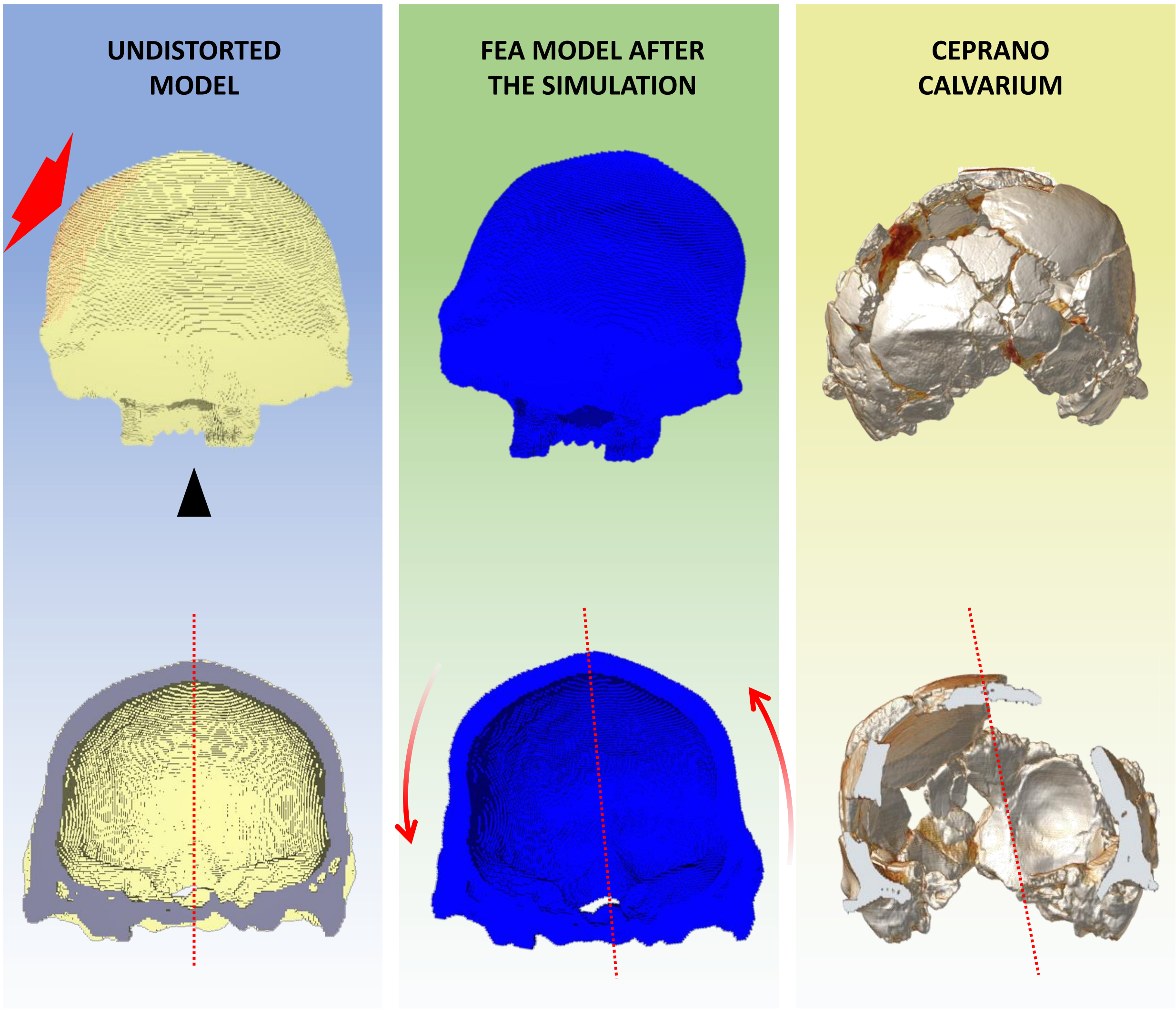

Figure S6. After the TPS-based analysis of the deformation recorded on the Ceprano calvarium (which suggested an affine pattern of deformation), we tested the hypothesis that such deformation resulted by the compressive forces operating on the specimen during the diagenesis. With this purpose, we have used a FEA modelization using the Petralona cranium as a proxy (first column, yellow). We have applied on the cranial vault a force of compression perpendicular to the surface and uniformly distributed on the left parietal (left column, arrow and reddish area); no forces were applied on the frontal nor on the occipital and temporal bone, while the constrain was on the base (black arrow). The resulting pattern of deformation (central column, blue) is similar to the observed condition in Ceprano (right column); rotation effect and inclination are emphasized.

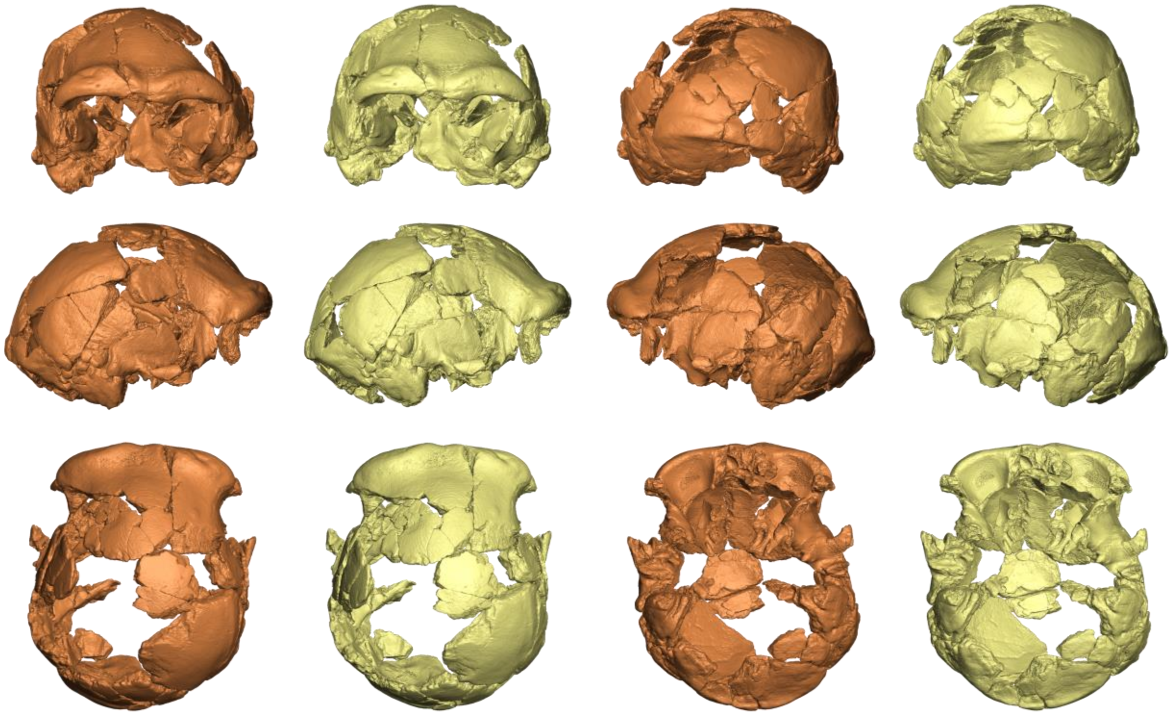

Figure S7 Our reconstruction after restoration (light brown) in comparison with its retrodeformed version (yellow); see the main text and Figure 7 for details.

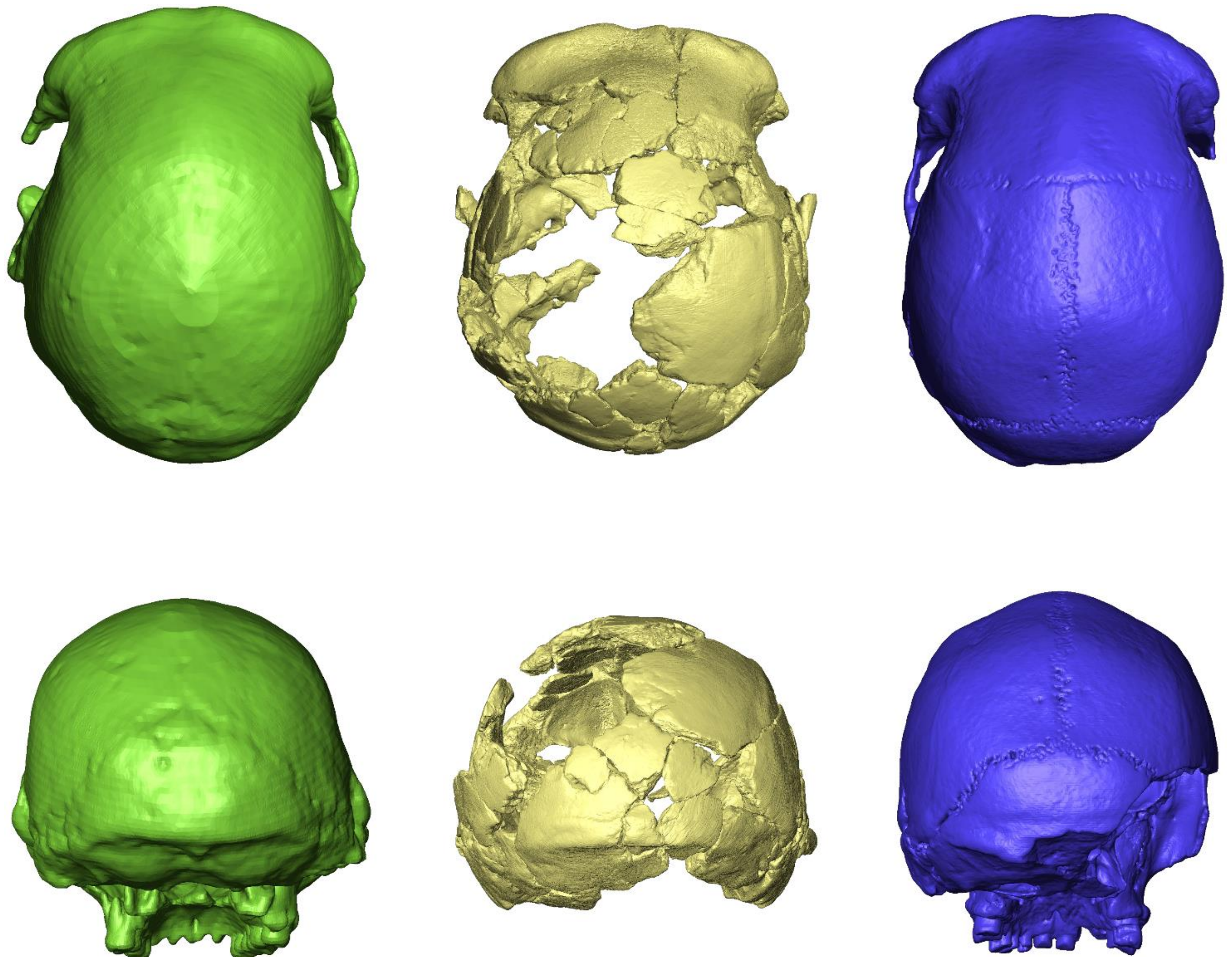

Figure S8 Comparison in superior view (top row) and posterior view (bottom row) between the reconstructed and retrodeformed calvarium of Ceprano (center, in yellow) and Mid-Pleistocene specimens from Petralona (left) and Kabwe (right). The crania are at the same scale and oriented according to the Frankfurt horizontal plane (approximated in Ceprano).
